# Impact of Plant Oil Supplementation on Lipid Production and Fatty Acid Composition in *Cunninghamella elegans* TISTR 3370

**DOI:** 10.3390/microorganisms12050992

**Published:** 2024-05-15

**Authors:** Surasak Khankhum, Karnjana Khamkaew, Hua Li, Chuenjit Prakitchaiwattana, Sirithon Siriamornpun

**Affiliations:** 1 Department of Biology, Faculty of Science, Mahasarakham University, Kantarawichai 44150, Maha Sarakham, Thailand; surasak.kk@msu.ac.th; 2 Senangkhanikhom School, Secondary Educational Service Area Office Ubonratchathani—Amnat Charoen, Senangkhanikhom 73290, Amnat Charoen, Thailand; khamkaewkarnjana@gmail.com; 3 Department of Cuisine and Nutrition, Yangzhou University, Yangzhou 225127, China; lihua216@yzu.edu.cn; 4 Department of Food Technology, Faculty of Science, Chulalongkorn University, Payatai, Patumwan, Bangkok 10330, Thailand; cheunjit.p@chula.ac.th; 5 Research Unit of Thai Food Innovation, Department of Food Technology and Nutrition, Faculty of Technology, Mahasarakham University, Kantarawichai 44150, Maha Sarakham, Thailand

**Keywords:** fungi, rice bran oil, unsaturated fatty acids, perilla oil, soybean oil

## Abstract

The *Cunninghamella* genus has been utilized for the production of PUFA-rich lipids. Therefore, we investigate the impact of plant oil supplementation in the culture medium (soybean oil, rice bran oil, and perilla oil), selected based on their different fatty acid predominant, on lipid production and fatty acid composition in *C. elegans* (TISTR 3370). All oils significantly boosted fungal growth, each influencing distinct patterns of lipid accumulation within the cells. The cells exhibited distinct patterns of lipid accumulation, forming intracellular lipid bodies, influenced by the different oils. Monounsaturated fatty acids (MUFAs) were found to be the most abundant, followed by polyunsaturated fatty acids (PUFAs) and saturated fatty acids (SFAs) in the fungal lipid cultures. Oleic acid was identified as the primary MUFA, while palmitic acid was the predominant SFA in perilla oil supplements. Remarkably, perilla oil supplement provided the highest total lipid production with arachidonic acid being exclusively detected. The percentage of PUFAs ranged from 12% in the control to 33% in soybean oil, 32% in rice bran oil, and 61% in perilla oil supplements. These findings offer valuable opportunities for advancing biotechnological applications in lipid production and customization, with implications for food and nutrition as well as pharmaceuticals and cosmetics.

## 1. Introduction

Fatty acids play a crucial role in various physiological functions within the human body and other organisms. The majority of naturally occurring fatty acids typically consist of an even number of carbon atoms, ranging typically from 14 to 22 atoms. Among these, fatty acids containing either 16 or 18 carbon atoms are particularly prevalent. Fatty acids are commonly classified into two primary types: saturated fatty acids (SFAs) and unsaturated fatty acids (UFAs). Unsaturated fatty acids further divide into monounsaturated fatty acids (MUFAs), such as oleic acid and palmitoleic acid, and polyunsaturated fatty acids (PUFAs), which encompass omega-3 (n-3 PUFAs), omega-6 (n-6 PUFAs), and omega-9 (n-9 PUFAs) fatty acid groups [1,2]. Among these, polyunsaturated fatty acids (PUFAs) play crucial roles in higher eukaryotes as energy stores and membrane constituents [3,4]. Their biological activities make them potential candidates for food additives or pharmaceuticals, with applications in the treatment of cardiovascular disorders, cancer, inflammatory reactions, liver lipid disorders, and postpartum mental health [1,5,6,7,8,9]. Unlike other organisms, humans are unable to synthesize PUFAs in sufficient quantities, necessitating their acquisition through dietary sources [10,11]. Seed oils and fish are currently the primary sources of PUFAs, although with limited yields and high manufacturing costs relative to the extracted fatty acid quantity [11,12].

Certain microorganisms from various sources, including bacteria, yeast, mold, and algae, have the ability to accumulate lipids comprising over 20% of their dry biomass, while oleaginous yeasts and molds can even reach over 70% lipid content [13,14,15,16]. The biosynthesis of PUFAs in microorganisms commences with the conversion of MUFAs, such as oleic acid, through enzymatic processes analogous to those found in higher organisms [17]. This process involves pivotal steps including elongation of the carbon chain facilitated by elongate enzymes and desaturation catalyzed by fatty acyl desaturases, leading to the incorporation of double bonds. Typically, the initial double bond is introduced at the Δ9 position of saturated fatty acids, yielding prevalent monoenes like palmitoleic (16:1 cis 9) and oleic (18:1 cis 9) acids. Subsequently, oleic acid undergoes desaturation by Δ12 desaturase to produce linoleic acid, which may further convert to α-linolenic acid via Δ15 desaturase. These MUFAs serve as precursors for the synthesis of n-9, n-6, and n-3 fatty acids. Further PUFA synthesis requires desaturation mediated by Δ6 desaturase of appropriate fatty acid precursors, followed by chain elongation and additional desaturation steps, ultimately yielding C20 and C22 PUFAs [2,15,16,17]. Among the fungi, species of the *Cunninghamella* genus, belonging to the oleaginous Zygomycetes, have been utilized for the production of PUFA-rich lipids such as γ-linolenic acid (GLA) [6,18,19,20]. The Mucoralean fungus *C. elegans* CFR C07 has emerged as a promising source of γ-linolenic acid (GLA), exhibiting a preference for glucose as the primary carbon source under submerged conditions, while fructose and sucrose have demonstrated efficacy in biomass production. Notably, GLA production reached a maximum of 882 mg/L in shake flasks and 733 mg/L in a 3 L fermenter [21]. Furthermore, the addition of vegetable oil to cultures enhanced GLA production by *C. elegans* CCF 1318, yielding 14.2 mg of GLA per gram of dry substrate, composed of a mixture of barley, spent malt grains, and peanut oil [22]. Notably, the fat1 gene in *C. elegans* encodes a novel omega-3 desaturase capable of introducing a double bond at the omega-3 position of C18 and C20 fatty acids [23].

Plant seed oils contain a diverse array of crucial fatty acids, varying depending on the plant species and extraction technique employed. These oils are rich sources of saturated, monounsaturated, and polyunsaturated fatty acids, including linolenic acid and linoleic acid, essential for human nutrition [24]. Additionally, n-9 and n-6 fatty acids are commonly present in plant seed oils, playing significant roles in cellular health and overall wellbeing [1,5,9,24]. Rice bran oil, extracted from the outer layer of rice bran, is predominantly composed of unsaturated fatty acids, constituting approximately 76% of its composition. This includes essential linoleic acid (n-6) and α-linolenic acid (n-3) at concentrations of up to 31.7%, along with oleic acid at 30–40%. Furthermore, it contains vitamins E and B, as well as antioxidant compounds such as tocols (tocopherols and tocotrienols) [25]. Soybean oil, in addition to its high oleic and linoleic acid content, contains beneficial fatty acids, with linolenic acid comprising approximately 50%, oleic acid 22%, and linolenic acid (C18:3) at 7%, alongside palmitic and stearic acids at 7% [26]. Perilla oil is predominantly composed of unsaturated fatty acids, particularly high in α-linolenic acid, with a fatty acid composition ratio of saturated, monounsaturated, and polyunsaturated fatty acids at 1:1:8. Major PUFAs in perilla oil include alpha-linolenic acid (18:3, n-3) at 55–60%, linoleic acid (18:2, n-6) at 18–22%, and oleic acid (18:1) at 11–13% of total fatty acids [27].

By utilizing exogenous fatty acids as alternative carbon sources, fungi can incorporate and modify the composition of accumulated lipids, reflecting the carbon chain length and structure of the oil used [27,28]. In previous studies, the addition of plant oils as precursors to fungi (*Mortierella alpine*), black yeast (*Melanodevriesia melanelixiae*), and growth media has been demonstrated to increase the production of PUFA [28,29,30]. However, no such investigation has been conducted in *C. elegans*, particularly concerning different fatty acid patterns. Moreover, there is limited information available regarding plant oil supplements that promote fungal cell growth and PUFA production. These supplements encompass plant oils containing diverse predominant PUFAs, such as oleic acid (rice bran oil), linoleic acid (soybean oil), and α-linolenic acid (perilla oil). Consequently, the objective of this study was to examine the effects of various plant oils added to the medium on *C. elegans* fungal lipid production and fatty acid composition. We anticipated obtaining valuable insights for subsequent applications in lipid production and customization, with implications for food and nutrition, pharmaceuticals, and cosmetics.

## 2. Methods

### 2.1. Microorganism, Culture Medium, and Growth Conditions

*Cunninghamella elegans* TISTR 3370, a fungus, was obtained from the Thailand Institute of Scientific and Technological Research (TISTR). To maintain the cultures, Sabouraud agar (HiMedia Laboratories Pvt. Ltd., Mumbai, India) was utilized and stored at 4 °C. For lipid production, *C. elegans* was cultivated in a semi-synthetic liquid medium containing the following components per liter: KH_2_PO_4_ (2.5 g), ZnSO_4_·7H_2_O (0.01 g), CuSO_4_·5H_2_O (0.002 g), MnSO_4_ (0.01 g), MgSO_4_·7H_2_O (0.5 g), FeSO_4_·7H_2_O (0.02 g), CaCl_2_ (0.1 g), yeast extract (5.0 g), KNO_3_ (1.0 g), and glucose (30 g), adjusted to pH 5.5. Before adding it to the culture medium, a mixture of oil and Tween 20 (0.5%) was prepared. The medium was supplemented with three different oils (15 g/L): soybean oil, rice bran oil, and perilla oil.

Sporangioles of *C. elegans* were collected from cultures grown on PDA (HiMedia Laboratories Pvt. Ltd., Mumbai, India) for seven days at room temperature. Ten 1 cm diameter mycelial disks from stock cultures on PDA plates were inoculated into the media. The cultures were then transferred to 250 mL Erlenmeyer flasks containing 50 mL of the semi-synthetic medium supplemented with each oil. Incubation was carried out at 25 °C, with shaking at 150 rpm, for eight days. Transmission electron microscopy (TEM) was employed to investigate cell morphology, lipid body number, and size, as well as to analyze lipid accumulation. Biomass, lipid accumulation in cells, and the fatty acid profile were determined, with duplicate analyses performed every two days throughout the cultivation period.

### 2.2. Transmission Electron Microscopy (TEM) Assay

To prepare the cultivated cells of *C. elegans* for transmission electron microscopy (TEM), a modified version of the protocol described by Jeh et al. [31] was followed. The mycelium was collected from the culture and subjected to centrifugation at 10,000× *g* at 4 °C for 10 min. The resulting cell pellet was immediately immersed in 3% glutaraldehyde at room temperature for 24 h for chemical fixation. Following fixation, the cells were washed three times with 1 M phosphate buffer and subsequently postfixed with 1% osmium tetroxide (OsO_4_) at 4 °C for 2 h. Another three washes were performed using the same buffer. Dehydration of the cells was carried out by sequential immersion in 70% ethanol for 10 min, followed by 100% ethanol for an additional 10 min. The cells were then embedded in spur resin. Ultrathin sections of approximately 70 nm were obtained and mounted on copper grids. Staining was conducted using 2% uranyl acetate and lead stain solution (Sigma-Aldrich, Inc., St. Louis, MO, USA) in a consecutive manner. Finally, the samples were observed using a transmission electron microscope (JEOL Ltd., Tokyo, Japan, JEM-1200).

### 2.3. Dry Biomass Inspection

The mycelia were collected from 50 mL samples and underwent two washes with distilled and deionized water using filter paper for filtration. Subsequently, the harvested mycelia were gently dried at 80 °C and placed in a vacuum desiccator until reaching a constant weight, indicating biomass stability. The dried mycelia were then prepared for lipid acid profile analysis, as described in the subsequent sections.

### 2.4. Lipid Extraction

Lipids were extracted from the desiccated mycelia following the method outlined by Bligh and Dyer [32], with some modifications. Approximately 3 g of finely ground samples were mixed with 50 mL of chloroform-methanol (2:1, *v*/*v*) containing 10 mg/L of butylated hydroxytoluene and 0.1 mg/mL of nanodecanoic acid (C19:0, Sigma-Aldrich, Inc., St. Louis, MO, USA) as an internal standard. The samples were stored overnight in a fume enclosure. Afterward, each sample was filtered and transferred to a separate funnel, followed by the addition of 15 mL of 0.9% sodium chloride. The phases were separated by vigorous shaking, and the lower phase was evaporated and transferred to a 10 mL volumetric flask.

### 2.5. Fatty Acid Analysis

This analysis was performed using the method of Liu et al. [33], with some modifications. Transesterification using H_2_SO_4_ (0.9 mol/L in methanol) was employed to convert the total lipid extract into fatty acid methyl esters (FAMEs). FAMEs were filtered through a Sep-pak silica column before injection into the gas chromatograph. A Shimadzu model GC-2014 system equipped with flame ionization detection and a fused silica capillary column (DB-Wax, USA; 30 m × 0.25 mm, 25 m film thickness) was used for quantitative analysis. Nitrogen served as the carrier gas, and the temperature programming ranged from 150 °C to 230 °C. The emergent peaks were identified by comparing their retention time with internal standard fatty acid nanodecanoic acid (C19:0). Lipid content, fatty acid composition and concentration were calculated as the following formulas.

$$\mathrm{Fatty}\;\mathrm{acid}\;\mathrm{composition}=[\frac{\mathrm{area}\;\mathrm{under}\;\mathrm{each}\;\mathrm{peak}}{\mathrm{total}\;\mathrm{areas}\;\mathrm{of}\;\mathrm{all}\;\mathrm{fatty}\;\mathrm{acids}\;\mathrm{appeared}\;\mathrm{in}\;\mathrm{the}\;\mathrm{chromatogram}}]\times 100$$

$$\mathrm{Fatty}\;\mathrm{acid}\;\mathrm{composition}=[[\frac{\mathrm{area}\;\mathrm{under}\;\mathrm{each}\;\mathrm{peak}}{\mathrm{area}\;\mathrm{of}\;\mathrm{internal}\;\mathrm{standard}}]\times 100]\times 10/g\;\mathrm{sample}$$

### 2.6. Statistical Analysis

All statistical analyses were performed in triplicate using the XLSTATTM software for Windows (https://www.xlstat.com/en/, accessed on 18 January 2024). One-way analysis of variance (ANOVA) was conducted to assess differences, with a significance level set at *p* < 0.05. Tukey’s test was utilized for mean comparisons.

## 3. Results and Discussion

### 3.1. Impact of Plant Oils on Growth Profile and Lipid Content in C. elegans Cells

**Figure 1:**
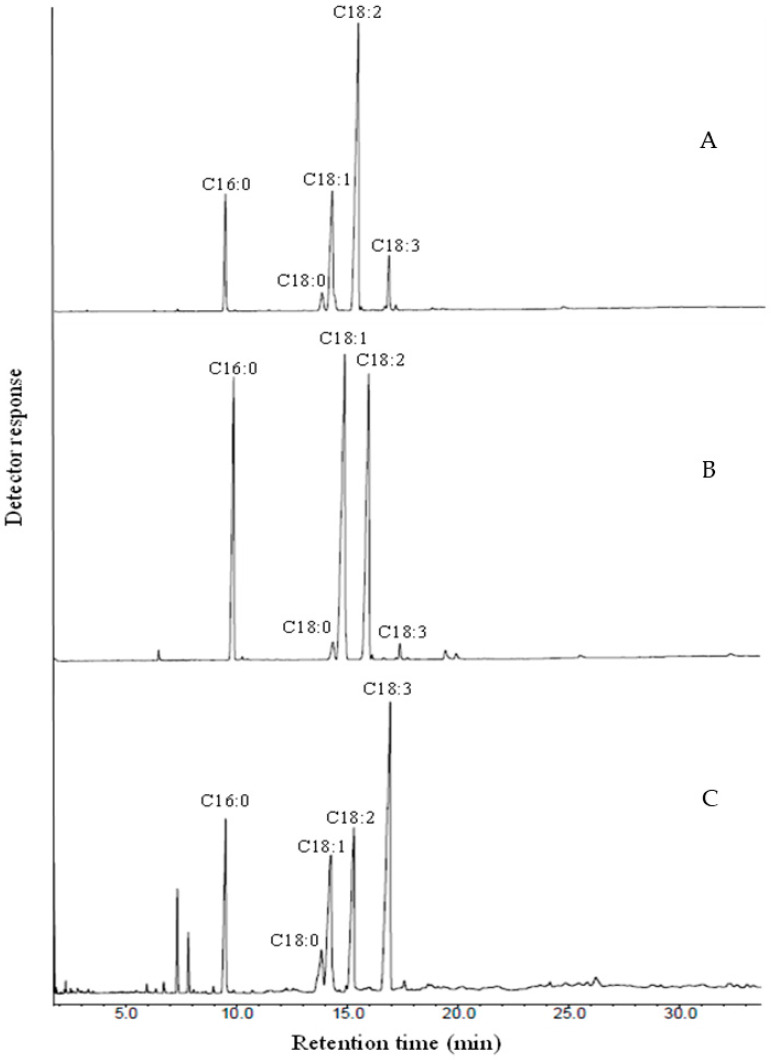
Chromatograms of fatty acids using different substrates. (**A**) Soybean oil, (**B**) rice bran oil and (**C**) perilla oil.

This experiment aimed to evaluate the effects of three different plant oils (soybean, rice bran, and perilla) when added to standard media (basal media) on the lipid content and fatty acid composition of *C. elegans* cells. The fatty acid compositions of the three oils were analyzed using gas–liquid chromatography (GLC) and are presented in Figure 1. Each oil exhibited a distinct fatty acid composition, with soybean oil being rich in 18:2n-6, rice bran oil dominated by 18:1, and perilla oil containing significant amounts of 18:3n-3, as shown in Figure 1. By supplementing fungal growth media with various oils, the intention was to provide precursors for long-chain fatty acids. Instead of synthesizing these fatty acids from scratch, fungi can modify the ones supplied through supplementation.

The growth profile of *C. elegans* cultivated in different plant oils is depicted in Table 1. The biomass production gradually increased with cultivation time, reaching the stationary phase by approximately day 6. The maximum cell growth of *C. elegans* occurred during the early growth phase, and the highest biomass content was observed as 14.15 ± 0.14 g/L and 27.67 ± 0.35 g/L on the 6th day for soybean and rice bran oils supplemented media, respectively, while for the perilla oil supplementation, the highest biomass occurred as 21.32 ± 0.19 g/L on day 8. Based on the total biomass obtained throughout the cultivation period, rice bran supplementation resulted in the highest biomass production, followed by perilla and soybean oil, respectively; all of which were significantly higher than that in the normal media. A study by Khoomrung et al. [34] that investigated the effect of exogenous fatty acids on *Mucor rouxii* cell growth, reported that fatty acid supplementation led to increased biomass, with the highest biomass was observed when linoleic acid (18:2n-6) was used.

Typically, carbohydrates are metabolized through the Embden–Meyerhoff pathway to produce pyruvate or acetyl-CoA, which are then utilized for protein synthesis, respiration, and the synthesis of various compounds, including membrane and storage lipids. In contrast, oil utilization by microorganisms involves the production of extracellular lipases that release fatty acids from glycerol. The resulting fatty acids can either be incorporated into lipid structures or broken down into basic substrates for biomass synthesis [35]. In line with this, previous studies, such as the one conducted by Dyal et al. [10], reported lower dry biomasses in supplemented media compared with unsupplemented media, with canola and olive oils resulting in the highest biomasses.

Interestingly, in our study, the fungus exhibited significantly higher growth rates in perilla oil compared with soybean and rice bran oils for up to 4 days (Table 1). This suggests that perilla oil might contain certain factors that stimulate cell division and/or enhance the production of cell wall components during the logarithmic growth phase. The lipid content of cells in each condition corresponded to their respective growth profiles. Perilla oil displayed the highest lipid yields from days 2 to 6 (23.33 ± 0.21, 24.33 ± 0.11, and 24.29 ± 0.57 g/L, respectively), followed by soybean oil on day 8 (20.61 ± 0.44 g/L), and rice bran oil (17.42 ± 1.67 g/L) had the lowest yields (Table 1). The lipid content reached its peak during the stationary phase, when nitrogen sources in the medium became depleted, preventing further lipid production. This finding aligns with previous reports where lipid accumulation ceased after 6 days in cultures of *Mucor* sp.1b and *M. rouxii* [36], although exceptions have been observed in some strains like *M. circinelloides* [37]. The decrease in malic enzyme activity following nitrogen exhaustion for approximately 24 h could explain the decline in lipid accumulation [15]. Oleaginous fungi have the ability to store lipids as reserves [38], and this accumulation occurs when the nitrogen source is depleted, restraining cell proliferation and allowing the substrate to be converted into lipids. Bajpai et al. [39] have reported maximum production of *M. alpina* IS-4 and ATCC 32222 after 3 and 4 days of incubation, respectively. Furthermore, Kendrick and Ratledge [40] noted that *M. isabellina* did not grow on media containing linseed oil, but the medium supplemented with safflower oil resulted in the highest cell yield, while perilla oil supplementation led to significantly lower cell yields compared with glucose medium alone. Regarding the lipid content of *M. isabellina*, safflower oil and perilla oil-supplemented media yielded the highest lipid content [40]. Previous studies have identified several additional variables that could potentially impact the lipid biosynthesis of fungi. Notably, antioxidants, a high proportion of protein, carbohydrates, and various minerals inherent in rice bran, soybean, and sesame oils have been implicated in this regard [41,42,43]. These factors have demonstrated a significant influence on the lipid production of Oleaginous *Mucor circinelloides* and *Schizochytrium limacinum* SR21 [44,45].

The findings of this study clearly demonstrate that plant oils have a substantial impact on enhancing the growth rate of *C. elegans* compared with the basal medium. Moreover, the increase in cell dry mass corresponded with higher lipid content. It is worth noting that the growth profile of the fungus and the lipid content varied significantly depending on the type of plant oil used [46]. Consequently, a detailed examination of cell morphology and lipid accumulation throughout the growth process was conducted to gain insights into the growth stage and cellular characteristics.

### 3.2. Lipid Accumulation in C. elegans during Growth on Oil Supplement Media

**Figure 2:**
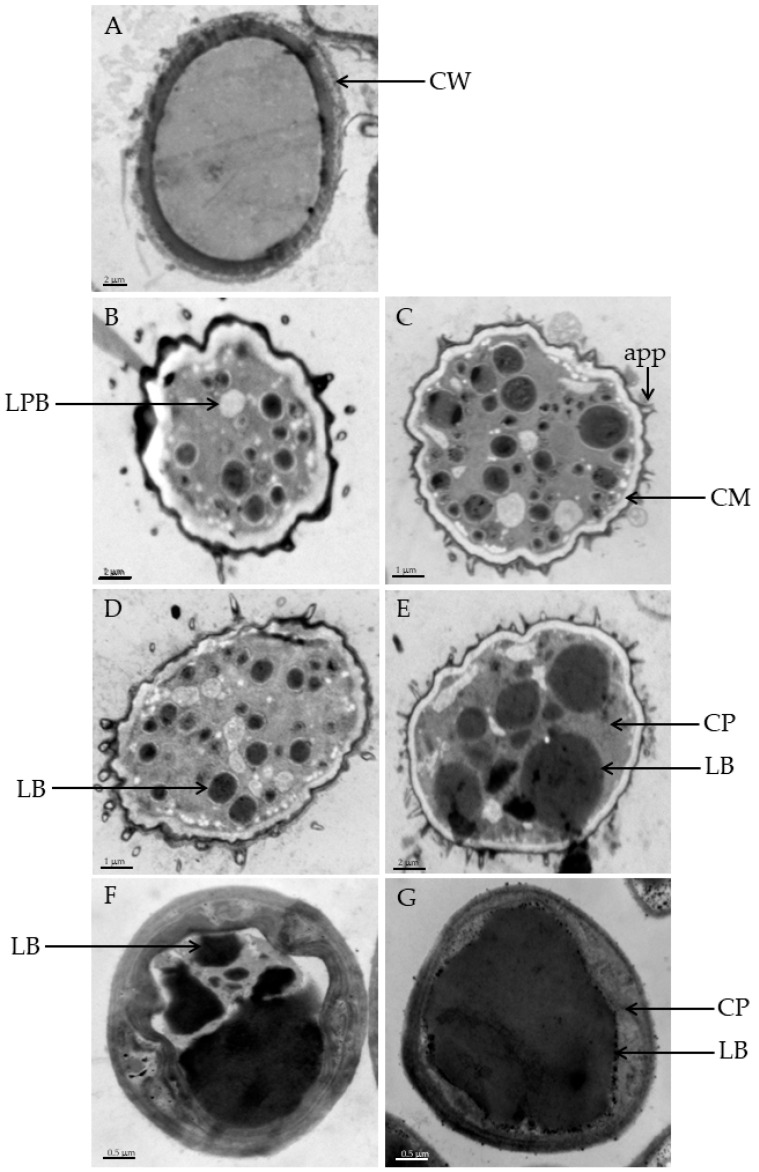
Transmission electron micrograph of *C. elegans* cells grown in soybean oil rice bran oil and perilla oil medium. TEM micrographs reveal lipids (dark spot) as an intracellular accumulation of lipid bodies. The characteristics of normal cells before the shift to soybean and rice bran oil-containing medium (**A**), cell grown in soybean oil at day 2 (**B**) and day 8 (**C**), cell grown in rice bran oil at day 2 (**D**) and day 8 (**E**) and cell grown in perilla oil at day 2 (**F**) and day 8 (**G**). LB, lipid-body; LPB, lipid-prebody; CP, cytoplasm; CW, cell wall; CM, cytoplasm membrane; app, appendages. Scale bar (**A**,**B**,**E**) = 2 μm, (**C**,**D**) = 1 μm and (**F**,**G**) 0.5 μm.

*C. elegans* cells were cultured in PDA medium until reaching the exponential growth phase. Subsequently, the cells were transferred to media supplemented with various oils. The observation of lipid bodies (LBs) in terms of their numbers and sizes was carried out using TEM. Prior to oil supplementation, LBs appeared as black spherical structures on the intracellular surface following osmium staining (Figure 2). Cells cultivated in oil-supplemented media exhibited appendages, and the number of appendages increased over time. From day 2 to day 8, the cells displayed heterogeneity in terms of size, diameter, and appearance, ranging from 0.8 to 4.2 µm, while LB sizes ranged from 0.1 to 2.6 µm. Notably, cells from the perilla oil-supplemented medium were significantly smaller compared with the other two oils. Differences in the thickness of the cell wall and the width of the periplasmic space were observed in normal cells (Figure 2A) prior to oil supplementation. Upon transferring the cells to oil-supplemented media, intracellular structural differences were also observed (Figure 2B–G). The TEM images revealed approximately 8 LBs in soybean oil supplementation and over 10 LBs in rice bran oil supplementation. After day 8, the number of LBs increased to over 10 in soybean oil, while it decreased to less than 10 in rice bran oil, although the LBs in the latter were larger in size (Figure 2B–E). In contrast, the supplementation of perilla oil displayed a distinct pattern, characterized by a small presence of LBs on day 2, which then expanded to completely fill the cell space by day 8 (Figure 2F,G). These lipid accumulations in the form of LBs in the cytoplasm were confirmed by TEM, which indicated that small LBs were observed on day 2 after cell transfer to oil-supplemented media, and the number and size of LBs increased during growth, depending on the oil supplement. By day 8, LBs filled a significant portion of the cell after induction. The TEM analysis also confirmed that the observed structures in the cells were LBs with variations in lipid accumulation levels. Unsupplemented cells exhibited a few small LBs after transfer to oil supplementation (Figure 2A). Following two days of growth in oil-supplemented media, the presence of one to six large LBs and a few small LBs was observed in soybean and rice bran oil supplements (Figure 2B,D). In the rice bran oil supplement, four large LBs almost completely filled the cell by day 8 (Figure 2E). Previous studies have reported that eukaryotes retain triacylglycerols (TAGs) as spherical lipid particles with diameters ranging from 0.1 to 50 µm, depending on the species and cell types [47]. The formation of LBs begins with the coupling of wax ester synthase/acyl-CoA:diacylglycerol acyltransferase (WS/DGAT) to the cytoplasmic membrane, leading to the formation of small lipid droplets (SLDs) that remain associated with the membrane-bound enzymes. The coalescence of SLDs within lipid prebodies eventually results in the formation of mature LBs in the cytoplasm [47]. Comparing the intracellular LB numbers and sizes to cell biomass and lipid contents clearly revealed a correlation between LB size, cell biomass, and lipid content. Cells with higher growth rates and lipid content, particularly those supplemented with perilla and rice bran oils, exhibited larger intracellular LBs, especially in the case of perilla oil. This suggests that the cells could rapidly assimilate fatty acids from perilla oil to form mature LBs. The cell supplemented with perilla oil displayed the highest lipid content among all oil supplements at day 2, which remained constant thereafter, even though growth was still observed until day 6. These findings highlight the potential of *C. elegans* as a promising strain for fatty acid production, particularly for overexpressing polyunsaturated fatty acids (PUFAs). Therefore, the investigation and discussion of key fatty acids associated with the alteration of the polyunsaturated fatty acid (PUFAs) profile during cultivation will be presented in the next section.

### 3.3. Fatty Acid Profiles of Plant Oils and Fungal Lipids

The fatty acid profiles of plant oils and fungal lipids are presented in Figure 1 and Figures S1–S4. The relative proportions of fatty acids were determined by measuring peak areas and comparing retention times to FAME standards for identification. The profiles of unsupplemented and rice bran oil-supplemented media were nearly identical. However, oil-supplemented media exhibited seven new components: Unknown 3, Unknown 4, Unknown 5, Unknown 6, Unknown 7, Unknown 8, and C20:4 (ARA), which were absent in unsupplemented media. In contrast, perilla oil supplementation only resulted in one new component, C20:4 (ARA), not found in soybean oil supplementation, rice bran oil supplementation, or unsupplemented media. Therefore, a total of six new unknown components and one PUFA (C20:4 ARA) were introduced through fungal culture.

The fatty acid profiles of unsupplemented media for *C. elegans* on different days are depicted in Figure 1A. The major components were oleic acid (C18:1), linoleic acid (C18:2), and palmitic acid (16:0), accounting for approximately 38–49%, 1.5–27%, and 17–21% of the total fatty acids, respectively. The remaining fatty acids constituted smaller percentages. Figure 1B,C display the percentages of total saturated fatty acids (SFAs), total monounsaturated fatty acids (MUFAs), and total polyunsaturated fatty acids (PUFAs) in the lipids. Compared with soybean and rice bran oil supplementation, perilla oil supplementation extended the profile to include short-chain polyunsaturated fatty acids. The ratio of PUFAs to SFAs ranged from 14–24% to 31–61% of total fatty acids in this study. Rice bran oil supplementation exhibited the highest percentage of MUFAs (42–47% of total fatty acids) among soybean, perilla, and rice bran oil supplements. In soy oil-supplemented cells, the production of PUFAs increased steadily over a four-day period, followed by a slight decrease between days 6 and 8, and then stabilized thereafter. The addition of soybean oil led to an increase in the unidentified product value, while the value of PUFAs decreased slightly.

### 3.4. Fatty Acid Composition and Concentration

The fatty acid composition and concentration of *C. elegans* under different oil supplements are presented in Table 2 and Table 3. The analysis included total saturated fatty acids (SFAs), total monounsaturated fatty acids (MUFAs), total polyunsaturated fatty acids (PUFAs), and unknown components (Table 2). The percentage of total SFAs ranged from 29% to 38% in unsupplemented media and soybean oil, while total MUFAs varied from 40% to 50% in unsupplemented media and 42% to 47% in rice bran oil. The percentage of total unknown components ranged from 2.2% to 26% in soybean oil. PUFAs were the dominant category of fatty acids, accounting for 31% to 61% in perilla oil. The major MUFAs identified in *C. elegans* was oleic acid (C18:1), and the major PUFAs were linoleic acid (C18:2), linolenic acid (C18:3), arachidonic acid (C20:4), eicosapentaenoic acid (C20:5), and docosahexaenoic acid (C22:6). Notably, arachidonic acid was present in perilla oil-containing media but absent in unsupplemented media, soybean oil, or rice bran oil-containing media. Previous studies by Jang et al. [48] have also reported increased accumulation of arachidonic acid, eicosapentaenoic acid, and docosahexaenoic acid with the addition of linseed oil and sunflower oil.

The addition of linseed oil to the culture medium of *M. ramanniana* species resulted in higher proportions of EPA and LA [10]. EPA production increased with increasing linseed oil concentration, regardless of the growth temperature. On the other hand, ARA content decreased as linseed oil concentration increased. MUFA was the most predominant fatty acid in the fungal oils analyzed, followed by PUFA and SFA. The concentration of total MUFAs ranged from 6152.12 to 6716.23, 4497.98 to 5767.50, and 4789.37 to 5903.60 mg/100 g in unsupplemented media, soybean oil, and rice bran oil-containing media, respectively (Table 3). The total PUFA concentration, primarily linolenic acid, was highest in cultures grown in perilla-containing media, ranging from 2019.24 to 3519.50 mg/100 g, and accounting for 18% to 41% of total fatty acids. The concentration of total SFAs ranged from 1065.28 to 6009.74 mg/100 g (Table 3). The main SFA found in fungal oils was stearic acid, ranging from 3% of total fatty acids in rice bran oil-containing media to 20% in unsupplemented media, followed by palmitic acid, with composition ranging from 11% in perilla oil-containing media to 26% in soybean oil-containing media.

The addition of plant oils as precursors for desired fatty acid synthesis can increase PUFA production in fungi. However, the fatty acid compositions of the added oils have a significant effect on the types of fatty acids produced. In this study, supplementation with perilla oil resulted in high production of linolenic acid and arachidonic acid, followed by soybean oil and rice bran oil for EPA and docosahexaenoic acid production. Soybean oil supplementation increased the yield of linoleic acid. Rice bran oil supplementation led to the highest yields of oleic and linoleic acids as fungal oil. The addition of plant oil to the growth medium significantly enhanced the yield of ARA and EPA, as these fatty acid supplements served as precursors [49]. The oils used in the study had different fatty acid compositions, with rice bran oil having a major content of oleic acid, soybean oil rich in linoleic acid, and perilla oil abundant in linolenic acid. Supplementation with rice bran oil, soybean oil, and perilla oil stimulated the production of total PUFAs and individual PUFAs.

The nature of the oil substrate influenced PUFAs synthesis, with approximately 40% of the substrate oil being converted to fungal lipid. PUFAs are crucial cellular metabolites, and their yields depend on cell proliferation. However, due to cell lysis, PUFA production gradually decreased during prolonged cultivation. The optimal duration for PUFA yield varied depending on the fatty acid type, with α-linolenic acid and linoleic acid synthesized before arachidonic acid and eicosapentaenoic acid. Further investigation into these mechanisms is recommended in order to gain a deeper understanding of how medium composition influences the synthesis of diverse fatty acids in fungal lipids. Such insights could guide the development of strategies for producing lipid-based pharmaceuticals or bioactive compounds.

## 4. Conclusions

The findings of this study highlight the significant growth enhancement of *C. elegans* through the use of plant oils. Furthermore, the fungal lipids produced by this fungus were identified as a valuable source of fatty acids. Remarkably, alterations in the fatty acid composition of the cultivation media directly influenced the fatty acid composition of the fungal lipids. The supplementation of plant oils rich in short-chain PUFAs (18:1, 18:2, and 18:3) served as precursors for the synthesis of longer-chain PUFAs, including 20:5 and 22:6. These results align with previous research suggesting that the medium composition and the endoenzymes present in fungi play significant roles in determining the diverse PUFA compositions observed in fungal lipids. Additional research is needed to optimize the production of specific fatty acids and increase overall lipid yield by enhancing fungal growth and influencing lipid accumulation. These findings offer valuable insights into the idea of refining culture conditions to boost lipid yields. The development of such microbial platforms holds promise for upscaling industrial lipid production, providing a sustainable alternative to conventional sources such as vegetable oils or animal fats. The insights derived from this study present significant opportunities for advancing biotechnological applications in lipid production and customization across various sectors, including food, nutrition, pharmaceuticals, and cosmetics.

## Figures and Tables

**Table 1:** Time course of growth (A) and lipid content (g/100 g) (B) of *Cunninghamella* elegans grown in different oils supplemented media.

Days	Biomass (g/L)				Lipid Content (g/100 g)			
	Control	Soybean	Rice Bran	Perilla	Control	Soybean	Rice Bran	Perilla
2	3.24 ± 0.59 ^d^	6.74 ± 1.29 ^c^	8.02 ± 0.23 ^d^	10.76 ± 0.04 ^d^	13.54 ±0.09 ^c^	17.61 ± 0.04 ^c^	16.73 ± 0.73 ^a^	23.33 ± 0.21 ^b^
4	6.53 ± 0.38 ^c^	7.13 ± 0.08 ^c^	8.88 ± 0.24 ^c^	14.81 ± 0.34 ^c^	10.18 ± 0.38 ^d^	17.75 ± 0.30 ^c^	17.73 ± 0.69 ^a^	24.33 ± 0.11 ^a^
6	8.62 ± 0.66 ^a^	14.15 ± 0.14 ^a^	27.67 ± 0.35 ^a^	19.75 ± 0.40 ^b^	14.13 ± 0.86 ^b^	18.31 ± 0.05 ^b^	14.90 ± 0.67 ^b^	24.29 ± 0.57 ^a^
8	7.35 ± 0.24 ^b^	11.18 ± 0.26 ^b^	17.70 ± 2.76 ^b^	21.32 ± 0.19 ^a^	17.25 ± 0.24 ^a^	20.61 ± 0.44 ^a^	17.42 ± 1.67 ^a^	19.98 ± 0.19 ^c^

Data are expressed as the mean ± standard deviation of three replicates. Data with the same letters in the same column of each day with different superscripts are significantly different at *p* < 0.05.

**Table 2:** Fatty acid composition (% of total fatty acids) of *Cunninghamella elegans* grown with different oils supplement for 8 days.

	Fatty Acids					Proportion of Fatty Acid										
Days		Saturated Fatty Acid (SFA)				Monounsaturated Fatty Acid (MUFA)			Polyunsaturated Fatty Acid (PUFA)							
		C14:0	C16:0	C18:0	Total	C16:1	C18:1	Total	C18:2	C18:3	C20:4	C20:5	C22:6	Total	Unknown PUFA	%
2	UN	1.2 ± 0.03 ^a^	21.3 ± 0.29 ^a^	15.4 ±0.16 ^a^	37.8 ± 0.17 ^a^	1.6 ± 0.03 ^a^	38.1 ±1.08 ^c^	39.8 ± 1.05 ^a^	20.7 ±0.80 ^c^	0.1 ± 0.01 ^d^	ND	0.6 ± 0.02 ^c^	0.2 ± 0.02 ^c^	21.5 ± 0.83 ^d^	0.9 ± 0.05 ^d^	100
	SO	0.8 ± 0.01 ^b^	20.7 ± 0.24 ^b^	9.1 ± 0.04 ^b^	30.6 ± 0.27 ^b^	0.4 ± 0.01 ^b^	39.2 ± 0.05 ^b^	39.6 ± 0.06 ^a^	22.8 ± 0.23 ^b^	1.1 ± 0.01 ^b^	ND	1.3 ± 0.09 ^b^	1.1 ± 0.01 ^a^	26.2 ± 0.30 ^c^	3.6 ± 0.03 ^b^	100
	RO	0.4 ± 0.01 ^c^	20.1 ± 0.84 ^b^	2.8 ± 0.04 ^d^	23.4 ± 0.81 ^c^	0.3 ± 0.01 ^c^	41.5 ± 0.03 ^a^	41.8 ± 0.04 ^a^	30.8 ± 0.86 ^a^	0.8 ± 0.01 ^c^	ND	0.4 ± 0.01 ^c^	0.7 ± 0.04 ^b^	32.7 ± 0.92 ^b^	2.1 ± 0.13 ^c^	100
	PO	0.3 ± 001 ^d^	11.5 ± 0.27 ^c^	5.9 ± 0.06 ^c^	17.8 ± 0.33 ^d^	0.4 ± 0.01 ^bc^	23.1 ± 0.30 ^d^	23.5 ± 0.31 ^b^	13.3 ± 0.35 ^c^	31.1 ± 0.29 ^a^	3.3 ± 0.02	3.0 ± 0.01 ^a^	0.7 ± 0.01 ^b^	51.4 ± 0.68 ^a^	7.3 ± 0.03 ^a^	100
4	UN	0.9 ± 0.15 ^a^	17.9 ± 0.02 ^b^	9.8 ± 0.07 ^a^	28.6 ± 0.24 ^a^	1.3 ± 0.14 ^a^	38.4 ± 0.70 ^b^	39.7 ± 0.84 ^b^	26.6 ± 1.34 ^c^	1.1 ± 0.04 ^bc^	ND	0.6 ± 0.01 ^b^	1.7 ± 0.22 ^a^	30.1 ± 1.09 ^bc^	1.6 ± 0.02 ^c^	100
	SO	0.4 ± 0.01 ^b^	18.8 ± 0.17 ^a^	7.6 ± 0.10 ^c^	26.8 ± 0.27 ^b^	0.4 ± 0.03 ^c^	37.7 ± 0.88 ^b^	38.1 ± 0.85 ^b^	29.6 ± 0.50 ^b^	1.7 ± 0.06 ^b^	ND	0.9 ± 0.02 ^a^	0.8 ± 0.04 ^b^	32.9 ± 0.55 ^b^	2.2 ± 0.03 ^b^	100
	RO	0.4 ± 0.01 ^b^	18.6 ± 0.31 ^a^	2.6 ± 0.02 ^d^	21.7 ± 0.34 ^d^	0.5 ± 0.01 ^b^	43.6 ± 0.13 ^a^	44.1 ± 0.15 ^a^	30.5 ± 0.16 ^a^	0.7 ± 0.01 ^c^	ND	0.3 ± 0.01 ^c^	0.8 ± 0.01 ^b^	32.4 ± 0.15 ^b^	1.8 ± 0.15 ^c^	100
	PO	0.4 ± 0.02 ^b^	14.6 ± 0.60 ^c^	8.7 ± 0.35 ^b^	23.7 ± 0.97 ^c^	0.3 ± 0.02 ^c^	31.3 ± 0.99 ^c^	31.7 ± 1.04 ^c^	12.4 ± 0.54 ^c^	20.6 ± 0.17 ^a^	1.4 ± 0.03	0.4 ± 0.01 ^c^	1.3 ± 0.10 ^a^	36.0 ± 0.65 ^a^	8.6 ± 2.62 ^a^	100
6	UN	0.8 ± 0.02 ^a^	18.4 ± 0.19 ^b^	14.9 ± 0.25 ^a^	34.2 ± 0.45 ^b^	1.2 ± 0.05 ^a^	43.8 ±0.31 ^b^	45.1 ± 0.36 ^a^	16.8 ± 0.88 ^d^	0.4 ± 0.01 ^c^	ND	0.6 ± 0.01 ^b^	2.0 ± 0.04 ^a^	19.8 ± 0.83 ^d^	0.9 ± 0.01 ^c^	100
	SO	0.5 ± 0.02 ^b^	25.9 ± 0.19 ^a^	11.1 ± 0.13 ^b^	37.5 ± 0.30 ^a^	0.2 ± 0.01 ^c^	34.7 ± 0.78 ^c^	34.9 ± 0.78 ^b^	20.6 ± 1.16 ^b^	0.7 ± 0.01 ^b^	ND	1.0 ± 0.05 ^a^	1.0 ± 0.02 ^b^	23.3 ± 1.24 ^c^	4.3 ± 0.02 ^a^	100
	RO	0.4 ± 0.01 ^b^	18.9 ± 0.20 ^b^	6.0 ± 0.05 ^c^	25.4 ± 0.95 ^c^	0.6 ± 0.02 ^b^	46.2 ± 0.05 ^a^	46.8 ± 0.07 ^a^	23.0 ± 0.10 ^a^	0.2 ± 0.01 ^c^	ND	0.7 ± 0.02 ^b^	1.8 ± 0.01 ^b^	25.7 ± 0.14 ^b^	2.1 ± 0.01 ^b^	100
	PO	0.3 ± 0.01 ^c^	10.9 ± 0.01 ^c^	3.1 ± 0.02 ^d^	14.3 ± 0.04 ^d^	0.7 ± 0.02 ^b^	23.3 ± 0.02 ^d^	23.9 ± 0.02 ^c^	18.6 ± 0.01 ^c^	40.9 ± 0.02 ^a^	0.1 ± 0.01	0.3 ± 0.00 ^c^	0.9 ± 0.01 ^c^	60.9 ± 0.05 ^a^	0.9 ± 0.01 ^c^	100
8	UN	0.9 ± 0.02 ^a^	16.6 ± 0.63 ^b^	19.6 ± 0.66 ^a^	37.2 ± 1.39 ^a^	0.7 ± 0.05 ^a^	49.2 ± 1.19 ^a^	49.9 ± 1.24 ^a^	11.5 ± 0.11 ^b^	0.3 ± 0.06 ^c^	ND	0.1 ± 0.01 ^c^	0.6 ± 0.02 ^b^	12.5 ± 0.05 ^d^	0.4 ± 0.00 ^d^	100
	SO	0.4 ± 0.01 ^b^	18.3 ± 0.03 ^a^	8.2 ± 0.06 ^b^	26.9 ± 0.10 ^b^	0.5 ± 0.01 ^c^	32.6 ± 0.02 ^c^	33.1 ± 0.03 ^c^	11.9 ± 0.09 ^b^	0.3 ± 0.01 ^c^	ND	0.9 ± 0.04 ^a^	1.4 ± 0.04 ^a^	14.7 ± 0.18 ^c^	25.3 ± 0.27 ^a^	100
	RO	0.3 ± 0.01 ^c^	16.8 ± 0.22 ^b^	2.7 ± 0.01 ^d^	19.9 ± 0.20 ^c^	0.5 ± 0.01 ^b^	43.2 ± 0.10 ^b^	43.7 ± 0.11 ^b^	32.6 ± 0.09 ^a^	0.7 ± 0.01 ^b^	ND	0.3 ±0.01 ^b^	0.8 ± 0.01 ^b^	34.5 ± 0.12 ^a^	1.9 ± 0.01 ^c^	100
	PO	0.5 ± 0.01 ^b^	12.5 ± 0.05 ^c^	7.5 ± 0.07 ^c^	20.5 ± 0.13 ^c^	0.5 ± 0.01 ^c^	27.8 ± 0.62 ^d^	28.3 ± 0.63 ^d^	11.4 ± 0.14 ^b^	17.4 ± 0.67 ^a^	0.3 ± 0.01	0.3 ± 0.01 ^b^	1.4 ± 0.01 ^a^	30.9 ± 0.84 ^b^	20.3 ± 0.20 ^b^	100

ND, not detected; data are expressed as the mean ± standard deviation of three replicates. Data with the same letters in the same column of each day and with different superscripts are significantly different at *p* < 0.05. UN, no supplement; SO, soy oil supplement; RO, rice oil supplement; PO, perilla oil supplement.

**Table 3:** Fatty acid concentration (mg/100g) of *Cunninghamella elegans* grown with different oils supplement for 8 days.

	Fatty Acids					Concentration of Fatty Acid (mg/100 g)									
Days		Saturated Fatty Acid (SFA)				Monounsaturated Fatty Acid (MUFA)			Polyunsaturated Fatty Acid (PUFA)						
		C14:0	C16:0	C18:0	Total	C16:1	C18:1	Total	C18:2	C18:3	C20:4	C20:5	C22:6	Total	Unknown PUFA
2	UN	201.6 ± 9.43 ^a^	3562.1 ± 134.04 ^a^	2336.6 ± 140.62 ^a^	6009.7 ± 284.09 ^a^	295.6 ± 9.75 ^a^	5856.5 ± 12.13 ^a^	6152.1 ± 21.88 ^a^	4031.7 ± 158.61 ^a^	22.2 ± 0.97 ^d^	ND	80.9 ± 0.24 ^c^	25.8 ± 3.74 ^c^	4160.6 ± 161.56 ^b^	156.1 ± 12.37 ^d^
	SO	90.9 ± 1.64 ^b^	3174.8 ± 9.39 ^b^	1425.8 ± 11.87 ^b^	4691.5 ± 22.90 ^b^	77.6 ± 0.70 ^b^	5689.9 ± 11.45 ^a^	5767.5 ± 12.15 ^b^	2758.3 ± 221.46 ^c^	122.5 ± 0.90 ^b^	ND	167.3 ± 7.26 ^b^	137.8 ± 2.70 ^a^	3185.8 ± 231.86 ^d^	406.8 ± 2.76 ^b^
	RO	57.1 ± 0.14 ^c^	2492.4 ± 59.71 ^c^	353.8 ± 4.80 ^d^	2903.4 ± 64.65 ^c^	33.1 ± 0.03 ^c^	4756.2 ± 8.00 ^b^	4789.4 ± 8.03 ^c^	3614.9 ± 4.59 ^b^	96.0 ± 0.89 ^c^	ND	44.9 ± 0.22 ^d^	77.4 ± 4.82 ^b^	3833.2 ± 10.52 ^c^	239.3 ± 14.20 ^c^
	PO	33.8 ± 0.73 ^d^	1221.1 ± 31.73 ^d^	636.4 ± 7.73 ^c^	1891.3 ± 40.19 ^d^	39.9 ± 0.21 ^c^	1752.2 ± 4.41 ^c^	1792.1 ± 4.62 ^d^	1426.9 ± 33.36 ^d^	3306.0 ± 38.64 ^a^	351.27 ± 1.21	321.8 ± 0.76 ^a^	72.2 ± 0.03 ^b^	5478.1 ± 146.17 ^a^	772.1 ± 5.49 ^a^
4	UN	113.2 ± 2.45 ^a^	2398.8 ± 12.6 ^c^	1597.0 ± 13.92 ^a^	4020.3 ± 28.97 ^a^	217.9 ± 18.87 ^a^	6240.2 ± 13.77 ^a^	6458.2 ± 32.64 ^a^	4334.9 ± 288.28 ^a^	180.6 ± 3.93 ^c^	ND	91.4 ± 6.10 ^b^	263.9 ± 3.61 ^a^	4870.8 ± 301.92 ^a^	270.2 ± 1.60 ^c^
	SO	59.6 ± 2.82 ^b^	2811.5 ± 96.96 ^a^	1035.9 ± 5.20 ^b^	3907.1 ± 104.98 ^a^	57.9 ± 6.58 ^b^	5641.7 ± 11.25 ^b^	5699.7 ± 17.83 ^b^	4427.9 ± 187.98 ^a^	252.4 ± 15.14 ^b^	ND	132.5 ± 6.53 ^a^	113.9 ± 2.45 ^bc^	4926.7 ± 212.10 ^a^	321.6 ± 12.19 ^b^
	RO	55.5 ± 0.47 ^b^	2071.1 ± 9.15 ^d^	301.4 ± 0.52 ^c^	2428.2 ± 10.14 ^b^	67.1 ± 3.07 ^b^	4860.2 ± 8.35 ^c^	4927.4 ± 11.42 ^c^	3524.4 ± 102.19 ^b^	80.3 ± 0.47 ^d^	ND	37.2 ± 0.81 ^d^	91.7 ± 0.31 ^c^	3733.7 ± 103.78 ^b^	243.9 ± 1.47 ^d^
	PO	41.7 ± 0.20^c^	2526.3 ± 40.63 ^b^	1525.5 ± 45.93 ^a^	4093.7 ± 86.76 ^a^	33.5 ± 0.63 ^c^	5657.72 ± 11.32 ^b^	5691.2 ± 11.95 ^b^	1395.3 ± 1.67 ^c^	3079.3 ± 3.35 ^a^	139.25 ± 1.62	65.9 ± 3.40 ^c^	127.8 ± 13.89 ^b^	4807.5 ± 23.93 ^a^	851.9 ± 285.71 ^a^
6	UN	101.9 ± 5.29 ^a^	2840.9 ± 123.13 ^b^	1949.8 ± 22.18 ^a^	4892.7 ± 150.60 ^b^	161.85.55 ^a^	6389.8 ± 28.87 ^a^	6551.6 ± 34.42 ^a^	2185.6 ± 126.27 ^b^	50.3 ± 1.48 ^c^	ND	98.7 ± 2.15 ^b^	277.9 ± 31.66 ^a^	2612.4 ± 161.10 ^d^	115.8 ± 0.72 ^c^
	SO	75.4 ± 0.35 ^b^	34695.3 ± 57.94 ^a^	1484.8 ± 18.10 ^b^	36255.7 ± 76.39 ^a^	32.1 ± 1.47 ^d^	4645.8 ± 7.63 ^c^	4677.9 ± 9.10 ^c^	2591.6 ± 24.94 ^a^	88.9 ± 1.55 ^b^	ND	141.8 ± 10.00 ^a^	124.7 ± 0.97 ^c^	2946.9 ± 37.41 ^b^	574.5 ± 10.73 ^a^
	RO	46.8 ± 0.78 ^c^	2298.1 ± 94.00 ^c^	661.3 ± 14.95 ^c^	3006.3 ± 109.73 ^c^	72.7 ± 1.31 ^c^	5830.9 ± 24.04 ^b^	5903.6 ± 25.35 ^b^	2522.1 ± 49.14 ^a^	25.7 ± 0.72 ^d^	ND	75.3 ± 0.71 ^c^	193.5 ± 3.46 ^b^	2816.7 ± 54.03 ^c^	232.8 ± 2.33 ^b^
	PO	20.6 ± 0.02 ^d^	816.4 ± 0.83 ^d^	228.2 ± 0.72 ^d^	1065.9 ± 1.57 ^d^	90.8 ± 1.70 ^b^	2459.6 ± 25.77 ^d^	2550.4 ± 26.84 ^d^	2312.9 ± 74.04 ^b^	3519.5 ± 206.10 ^a^	9.80 ± 0.26	21.3 ± 0.02 ^d^	75.5 ± 0.49 ^d^	5939.1 ± 280.91 ^a^	71.7 ± 0.93 ^d^
8	UN	143.6 ± 22.37 ^a^	1983.7 ± 126.78 ^b^	3724.2 ± 100.44 ^a^	5851.7 ± 249.15 ^a^	82.7 ± 3.57 ^a^	6633.5 ± 15.56 ^a^	6716.2 ± 19.13 ^a^	1369.0 ± 23.41 ^c^	37.6 ± 8.50 ^d^	ND	13.3 ± 1.98 ^c^	67.6 ± 0.06 ^d^	1487.1 ± 33.82 ^d^	52.4 ± 1.65 ^d^
	SO	65.0 ± 4.33 ^c^	2350.1 ± 28.09 ^a^	1142.3 ± 43.27 ^b^	357.4 ± 75.69 ^d^	48.6 ± 0.43 ^c^	4449.4 ± 7.00 ^c^	4497.9 ± 7.43 ^c^	2082.7 ± 19.47 ^b^	53.1 ± 1.05 ^c^	ND	139.2 ± 2.79 ^a^	247.9 ± 6.96 ^b^	2522.9 ± 30.27 ^c^	4406.1 ± 40.88 ^a^
	RO	35.0 ± 1.47 ^d^	1867.1 ± 31.47 ^c^	324.4 ± 5.25 ^d^	2226.6 ± 68.71 ^c^	60.1 ± 0.51 ^b^	5057.5 ± 81.38 ^b^	5117.7 ± 81.89 ^b^	4083.0 ± 8.45 ^a^	92.1 ± 0.13 ^b^	ND	42.2 ± 0.98 ^b^	110.3 ± 1.91 ^c^	4327.6 ± 11.47 ^a^	142.1 ± 0.61 ^c^
	PO	98.2 ± 1.34 ^b^	1434.2 ± 11.90 ^d^	849.9 ± 6.47 ^c^	2382.4 ± 19.71 ^b^	51.8 ± 0.02 ^c^	3074.5 ± 3.34 ^d^	3126.4 ± 3.36 ^d^	1217.6 ± 12.56 ^c^	2019.2 ± 49.61 ^a^	69.32 ± 1.51	33.9 ± 0.16 ^b^	286.5 ± 2.75 ^a^	3626.6 ± 66.59 ^b^	4109.9 ± 42.80 ^b^

ND, not detected; data are expressed as the mean ± standard deviation of three replicates. Data with the same letters in the same column of each day and with different superscripts are significantly different at *p* < 0.05. UN, no supplement; SO, soy oil supplement; RO, rice oil supplement; PO, perilla oil supplement.

## Data Availability

Data are contained within the article and Supplementary Materials.

## Supplementary Materials

The following supporting information can be downloaded at: https://www.mdpi.com/article/10.3390/microorganisms12050992/s1, Figure S1: GC chromatogram of fatty acids for perilla oil supplemented media; Figure S2: GC chromatogram of fatty acids for soybean oil supplemented media; Figure S3: GC chromatogram of fatty acids for rice bran oil supplemented media; Figure S4: Chromatograms of fatty acids for unsupplement media.
