# Supplementary material for: Impact of Plant Oil Supplementation on Lipid Production and Fatty Acid Composition in Cunninghamella elegans TISTR 3370
Source: Microorganisms. 2024 May 15;12(5):992. doi: 10.3390/microorganisms12050992 (PMC11124419; doi:10.3390/microorganisms12050992)
Supplement: Supplementary file 1 [file microorganisms-12-00992-s001.zip › microorganisms-2994595-supplementary.pdf]

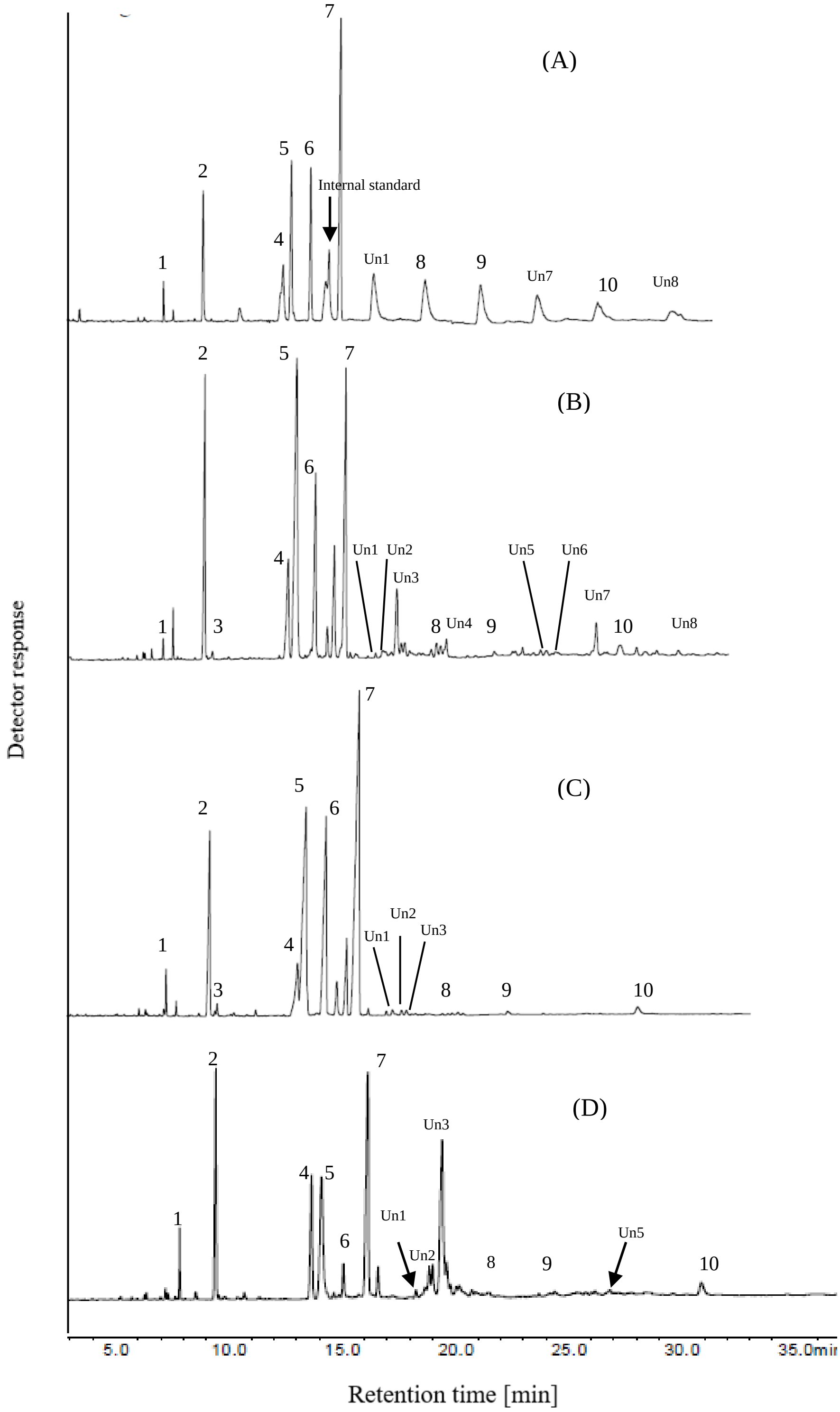

Figure S1 GC chromatogram of fatty acids for perilla oil supplemented media. Peak: (1)C14:0; (2) C16:0; (3)C16:1; (4)C18:0; (5)C18:1; (6)C18:2; (7)C18:3; (8)C20:4; (9)C20:5; (10)C22:6; (Un1)Unknown 1; (Un2)Unknown 2; (Un3)Unknown 3; (Un4)Unknown 4; (Un5)Unknown 5; (Un6)Unknown 6; (Un7)Unknown 7; (Un8)Unknown 8; (A)2day; (B): 4day; (C):6 day; (D) 8 day.

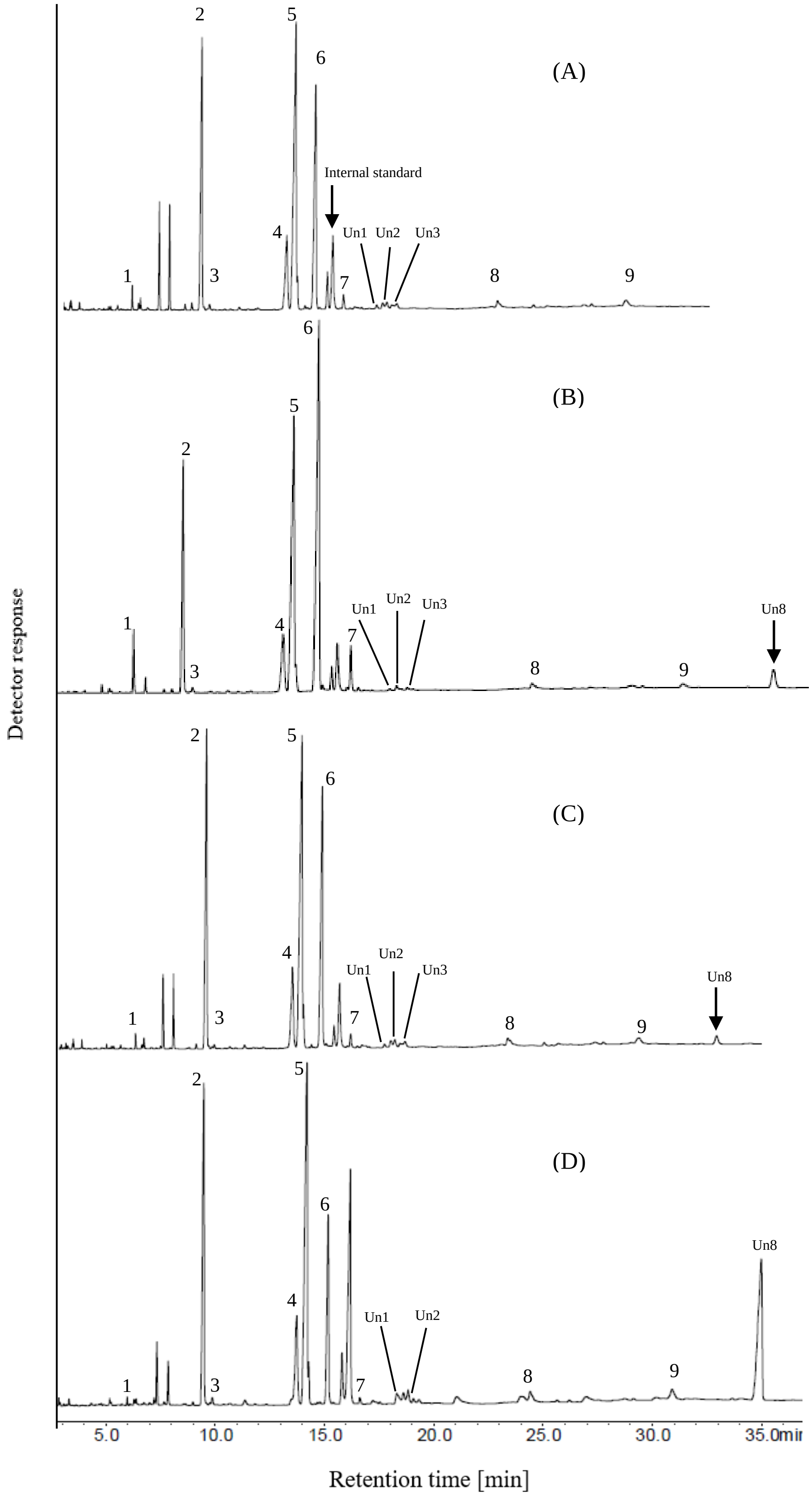

(A)
(B)
(C)
(D)
1
2
3
4
5
6
7
8
9
Internal standard
Un1
Un2
Un3
Un8
Detector response
5.0
10.0
15.0
20.0
25.0
30.0
35.0
Retention time [min]

Figure S2 GC chromatogram of fatty acids for soybean oil supplemented media. Peak: (1)C14:0; (2) C16:0; (3)C16:1; (4)C18:0; (5)C18:1; (6)C18:2; (7)C18:3; (8)C20:5; (9)C22:6; (Un1)Unknown 1; (Un2)Unknown 2; (Un3)Unknown 3; (Un4)Unknown 4; (Un5)Unknown 5; (Un6)Unknown 6; (Un7)Unknown 7; (Un8)Unknown 8; (A)2day; (B)4day; (C)6 day; (D)8 day.

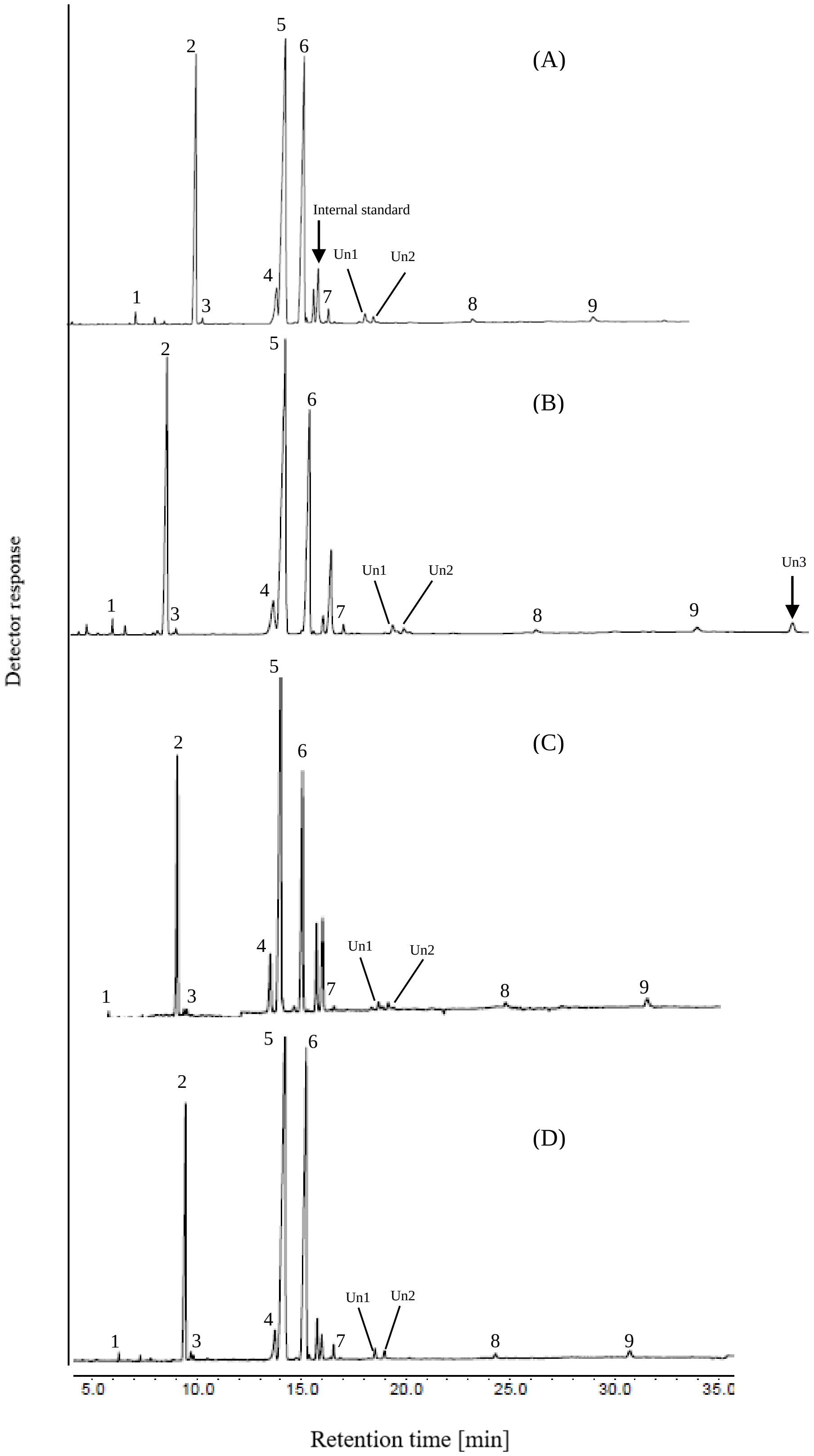

Figure S3 GC chromatogram of fatty acids for rice bran oil supplemented media. Peak: (1)C14:0; (2) C16:0; (3)C16:1; (4)C18:0; (5)C18:1; (6)C18:2; (7)C18:3; (8)C20:5; (9)C22:6; (Un1)Unknown 1; (Un2)Unknown 2; (Un3)Unknown 3; (A)2day; (B)4 day; (C)6 day; (D)8 day.

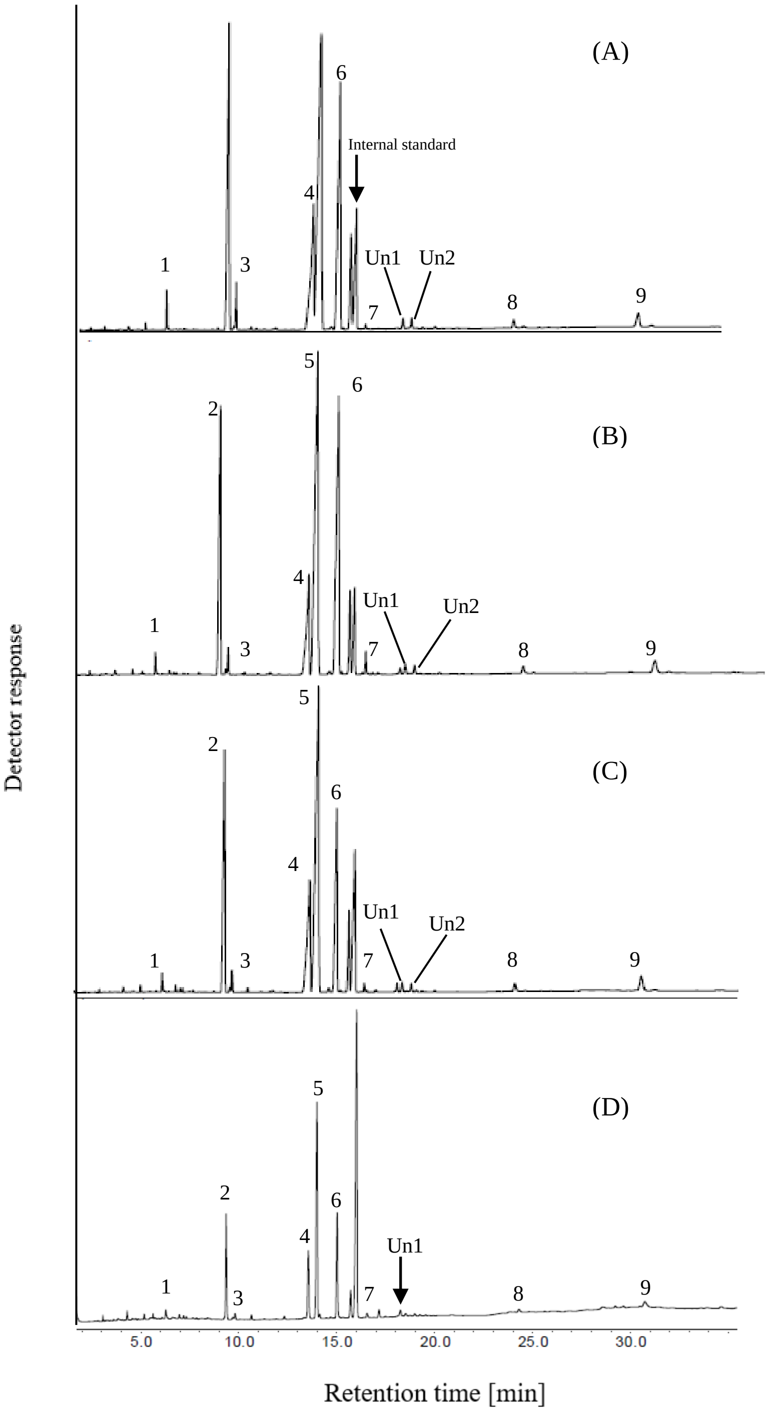

Figure S4 Chromatograms of fatty acids for unsupplement media. Peak: (1)C14:0; (2) C16:0; (3)C16:1; (4)C18:0; (5)C18:1; (6)C18:2; (7)C18:3; (8)C20:5; (9)C22:6; (Un1) Unknown 1; (Un2)Unknown 2; (A)2day; (B): 4day; (C)6 day; (D)8 day.
